# Case Report: PROS1 (c.76+2_76+3del) pathogenic mutation causes pulmonary embolism

**DOI:** 10.3389/fcvm.2024.1459579

**Published:** 2024-10-11

**Authors:** Peng Ding, Yuan Zhou, Meijie Yang, Sheng Li, Song Zhang, Lijia Zhi

**Affiliations:** ^1^ Department of Critical Care Medicine, Hospital of Chengdu University of Traditional Chinese Medicine, Chengdu, China; ^2^ Department of Geriatric Medicine, The General Hospital of Western Theater Command of PLA, Chengdu, China

**Keywords:** pulmonary embolism, PROS1, pathogenic mutation, protein S deficiency, case report

## Abstract

**Background:**

Genetic variation plays an extremely important pathogenic role in the development of venous thromboembolism (VTE). Genetic protein S (PS) deficiency caused by PROS1 gene mutation is an important risk factor for hereditary thrombophilia.

**Case introduction:**

In this case, we report a 28-year-old male patient who developed a severe pulmonary embolism during his visit. The patient had experienced one month of chest pains, coughing and hemoptysis symptoms. CTPA confirmed an acute pulmonary embolism with multiple filling defects in both pulmonary arteries. Ultrasound showed no thrombosis in the veins of both lower limbs. The patient's father and grandfather have a history of lower limb venous thrombosis. The patient was diagnosed with acute pulmonary embolism and pneumonia. The serum PS level significantly decreased (detection result: 10%, normal range: 77–143). Gene sequencing revealed a heterozygous missense mutation in PROS1 c.76+2_76+3del (base deletion), and further testing revealed that the genetic variation originated from his father. The patient was treated with heparin anticoagulant therapy, catheter thrombus aspiration, and catheter thrombolysis. After treatment, the patient's chest pain symptoms were relieved, and there were no symptoms such as difficulty breathing. On the 7th day of admission, the patient was transferred to a general hospital for further treatment.

**Conclusion:**

Hereditary thrombophilia caused by mutations in the PROS1 (c.76+2_76+3del) gene is extremely rare. In clinical practice, heparin and rivaroxaban treatment are beneficial.

## Introduction

Venous thromboembolism (VTE), including deep vein thrombosis (DVT) and pulmonary embolism (PE), is a major cause of unexpected death in patients and poses a serious threat to patient safety. PE is a common and potentially life-threatening cardiovascular condition. Its detection is often challenging due to the non-specificity of the patient's symptoms and signs (1). Although the 30-day mortality rate following pulmonary embolism has decreased, approximately 20% of patients still die before or shortly after diagnosis, particularly those with hemodynamic instability (2). The risk factors for PE can be categorized into hereditary and acquired. Acquired factors include long-term immobilization, major surgery, trauma, and certain medications. Genetic risk factors include deficiencies in protein S (PS), protein C (PC), antithrombin III deficiency, prothrombin gene mutations, and Leiden factor V (3). Hereditary PS deficiency is a rare autosomal dominant disorder caused by mutations in the PROS1 gene on chromosome 3, primarily presenting as venous thrombosis. Mutations in the PROS1 gene lead to alterations in the synthesis or function of PS. Based on differences in PS antigen levels and cofactor activity, PS deficiency can be classified into types I, II, and III. Studies have shown that individuals from families with mixed type I/III PS deficiency experience increased risks of hypercoagulable states and thrombosis in both type I and type III deficiencies. This indicates that both types (I and III) of PS deficiency impair anticoagulant function, thereby heightening the risk of venous thrombosis (4). Here we present a case of a young man hospitalized due to dyspnea and hypoxemia. Computerized Tomography Pulmonary Angiogram (CTPA) confirmed acute PE, and genetic sequencing identified a PROS1 missense mutation (c.76+2_76+3del).

## Case presentation

**Figure 1:**
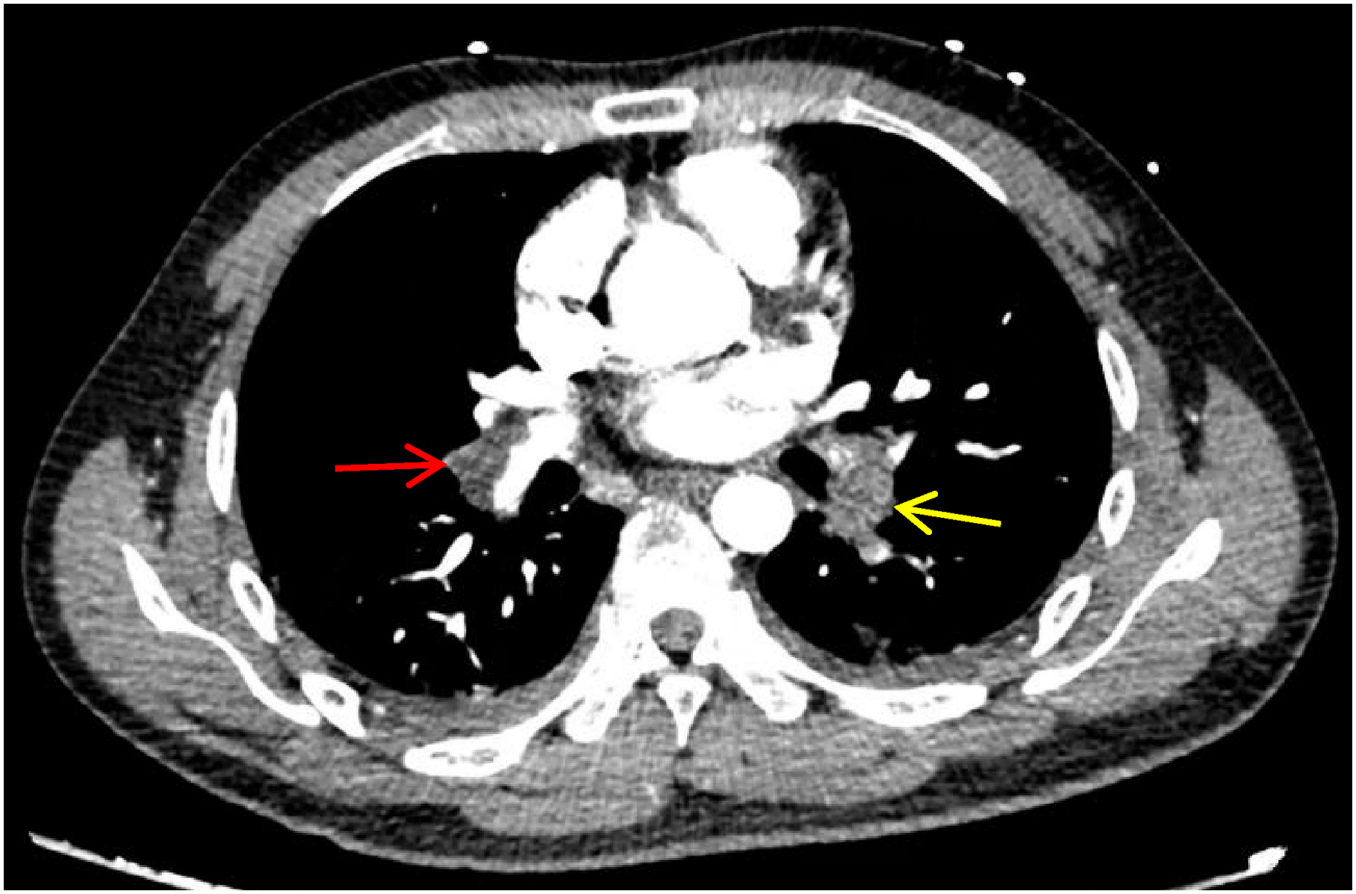
CTPA shows multiple pulmonary artery-filling defects in both lungs. The red arrow represents the main trunk of the right pulmonary artery, and the yellow arrow represents the lower left pulmonary artery.

A 28-year-old male patient, previously in good health, was admitted to the hospital due to a cough and chest pains. In August 2023, the patient developed a cough without any obvious cause, with bloody sputum. On September 8, 2023, he was admitted to a local hospital with a cough and right-sided chest pain. Blood tests revealed elevated D-dimer (5.79 mg/L, ↑) and whole blood C-reactive protein (48.28 mg/L, ↑). CTPA confirmed acute pulmonary embolism with multiple filling defects in both pulmonary arteries (Figure 1). He was subsequently diagnosed with acute pulmonary embolism and pneumonia after receiving alteplase (100 mg), morphine (10 mg), and cefmetazole (2 g every 8 h). Due to severe chest pain, breathing difficulties, and hypoxemia (SPO_2_ around 85% at rest), the patient was referred to our intensive care unit (ICU) on September 14, 2023. The patient's father and grandfather have a family history of lower limb venous thrombosis. He is neither obese nor sedentary, and he has no history of smoking or alcohol consumption.

After being transferred to the ICU, the patient received non-invasive ventilator-assisted breathing, using continuous positive airway pressure ventilation (pressure support: 10 cmH_2_O, oxygen concentration: 30%–60%, positive end expiratory pressure: 5 cmH_2_O), and intermittent high-flow oxygen inhalation (oxygen concentration: 30%–60%, airflow rate: 30–50 L/min). For anticoagulation heparin sodium was continuously infused at 750–1,250 units per hour, maintaining the activated partial thromboplastin time (APTT) at 60–80 s (normal range: 34–43). On September 15, 2023, the patient underwent pulmonary artery thrombus aspiration and thrombolytic therapy in the intervention room.

The surgical procedure was as follows. Following local anesthesia, the operator punctured the femoral vein and successfully inserted an 8 F sheath. A 0.035 mm diameter guidewire was used to introduce a 10 F long sheath into the pulmonary artery trunk. Angiography revealed filling defects in the right pulmonary artery trunk at the middle and lower lobe openings and in the distal branches. To address this, the operator repeatedly performed thrombus aspiration using a 10F thrombus aspiration catheter, whilst administering 100,000 units of urokinase as an adjunctive treatment. Follow-up angiography showed marked improvement in the filling defects of the right pulmonary artery trunk, with minimal residual defects and clear visualization of the distal branches. The procedure was completed successfully, and followed by sheath removal and effective compression for hemostasis.

**Figure 2:**
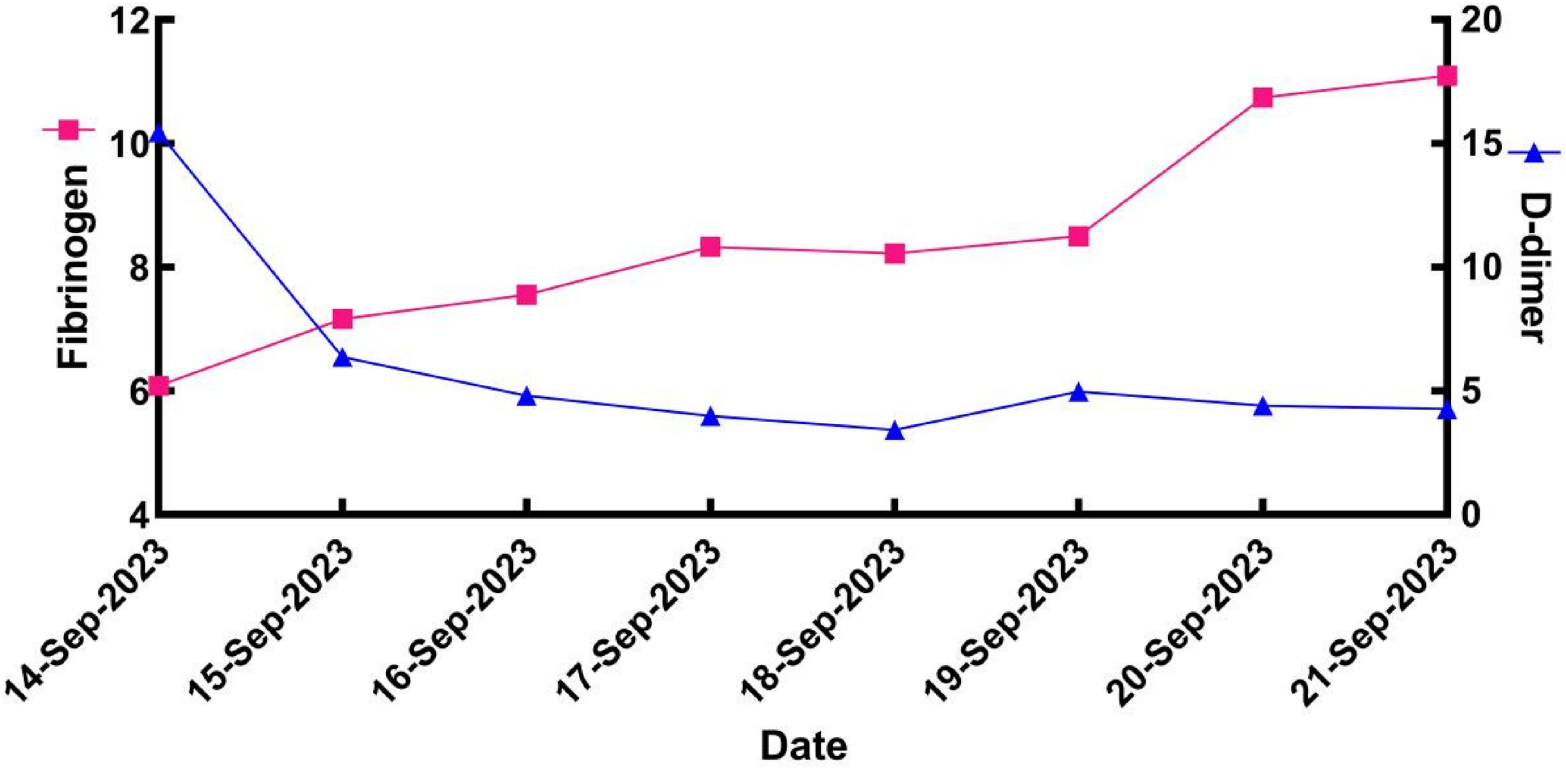
Fibrinogen and D-dimer levels of patients during ICU treatment.

After surgery, the patient continued to receive heparin sodium anticoagulant therapy, with regular monitoring of APTT, fibrinogen, and D-dimer levels (Figure 2). Testing for thrombotic disorders revealed a protein S level of 10% (normal range: 77–143 for males), while plasminogen (PLG), antithrombin III (AT-III), and protein C tests were negative.

### Follow-up

By the 7th day of admission, the patient's condition had improved and he was transferred to a local hospital for further treatment. The patient was treated with rivaroxaban (20 mg orally, once daily). After 6 months, his condition remained stable, with no recurrence of bleeding or thrombosis.

### Mutation site gene detection results and pathogenicity analysis

**Figure 3:**
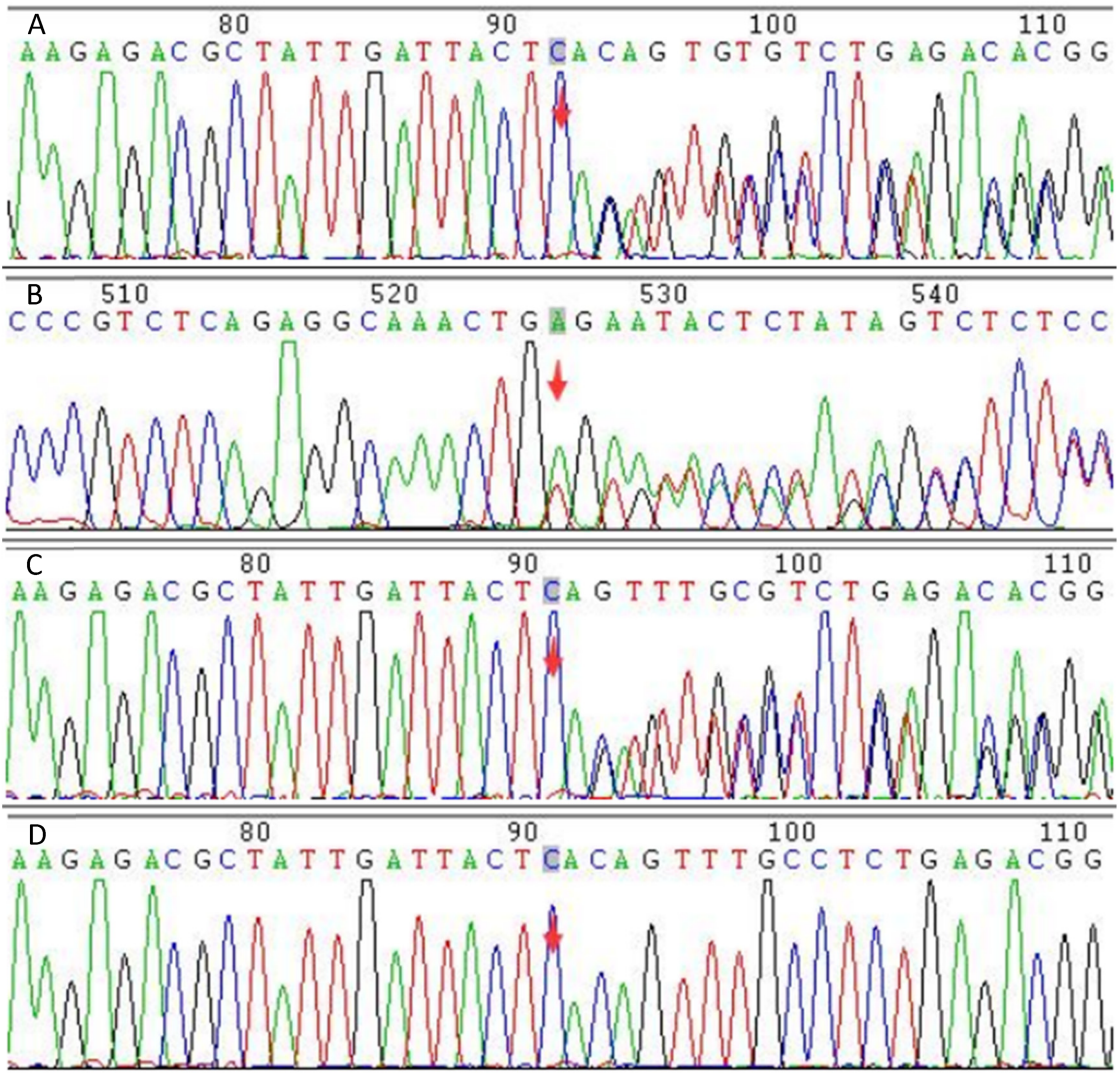
Figure 3 shows the Sanger sequencing results for the patient and their parents. **(A)** shows the forward sequencing of the patient's gene, **(B)** the reverse sequencing, **(C)** the forward sequencing of the father's gene, and **(D)** the forward sequencing of the mother's gene. The red arrow marks the mutation site. The sequence diagrams to the right of the arrow show a bimodal pattern in both the patient and the father, while the mother's sequence is unimodal. This indicates that the c.76+2_76+3del mutation, present in both the patient and the father, is a base deletion in the splicing donor region of the PROS1 gene. This mutation is predicted to cause mRNA splicing abnormalities.

Whole exome sequencing was used to analyze the gene coding regions in this case. The average sequencing depth was above 90X, with 98% of sequences exceeding 20X coverage. Genetic testing showed a heterozygous mutation (c.76+2_76+3del) in the 76th intron of the PROS1 gene on chromosome 3. Further analysis confirmed that the genetic variant was inherited from the patient's father (Table 1 and Figure 3). No abnormalities were detected in other thrombosis-related genes, including Jak-2 (myeloproliferative neoplasms), F2 (prothrombin G20210A), F5 (Factor V Leiden mutation), SERPINC1 (antithrombin III), and PROC (protein C). We used SpliceAI and Pangolin scores software (5) for pathogenicity prediction analysis, with a Donor Loss score of 0.98, indicating that the gene mutation is highly likely to cause it to lose its normal splicing function.

**Table 1:** Results of sanger verification.

Verify the site information	Relation	Sample test number	Verification result
PROS1 chr3:93692515_93692516	Patient	NP25F15780	Loss of heterozygosity
Intron:1/14	Father	VP25D10449	Loss of heterozygosity
NM_000313.4: c.76+2_76+3del	Mother	VP25D10448	Wild type

## Discussion and literature review

This section discusses a case of PROS1 (c.76+2_76+3del) gene mutation causing PS deficiency, which led to pulmonary embolism. The patient was treated with catheter thrombolysis, heparin, and rivaroxaban, resulting in an improvement to the patient's condition.

The PROS1 gene encodes PS, a protein that plays a crucial role in the human coagulation system. Mutations or deletions in this gene can cause functional abnormalities in PS, increasing the risk of thrombosis and other coagulation disorders. A large cohort study involving 140,214 participants from the UK Biobank investigated the impact of pathogenic variants related to genetic hemostatic disorders. It found that mutations in SERPINC1, PROC, and PROS1 significantly increased the risk of deep vein thrombosis and pulmonary embolism (6). Mutations in the PROS1 gene can occur in either coding exons or non-coding introns. Xu Fei et al. (7) reported heterozygous mutations occurring in the exon region, including c.458_458delA (p.Lys153Serfs*6), c.1687C>T (p.Gln563stop), and c.200A>C (p.Glu67Ala). Earlier studies have shown that intronic mutations often lead to abnormal RNA splicing, which was a common cause of PS deficiency in the UK thrombophilia cohort (8). In addition, multiple studies have found that intronic mutations, such as c.346+5G>C, c.602-2delA, and c.260-1G>A, lead to abnormal PS levels (9–11). In a previous study, we reported a PROS1 gene exon mutation (12). In this case, a deletion mutation occurred at the second and third base pairs of intron 76 in the PROS1 gene, resulting in impaired PS synthesis and subsequent pulmonary embolism.

Protein S is a multifunctional protein involved in various physiological processes, such as hemostasis, inflammation, and other cellular mechanisms. During the hemostasis process, PS acts as a cofactor for activated protein C (APC)-mediated protein hydrolysis by binding to APC, which is essential for effective APC-dependent coagulation regulation. Additionally, PS functions as a cofactor in the tissue factor pathway inhibitor (TFPI) pathway, regulating the extrinsic coagulation pathways (13). PS deficiency is closely associated with deep vein thrombosis. Hereditary PS deficiency is a rare but significant autosomal dominant genetic disorder, with deep vein thrombosis and pulmonary embolism being the main clinical manifestations in heterozygous patients (14). In clinical practice, thrombophilia testing is recommended for patients with a family history of venous thromboembolism and/or thrombophilia, in addition to for those with unexplained recurrent deep vein thrombosis (15). One of the key diagnostic steps is assessing plasma PS levels and activity. Patients diagnosed with hereditary PS deficiency usually require long-term anticoagulation therapy to prevent clot formation.

Pulmonary embolism is the third leading cause of cardiovascular death worldwide, following a stroke or heart attack. Severe pulmonary embolism cases often require advanced life support in the ICU. ICU interventions focus on oxygen delivery, fluid management, and catecholamine administration until pulmonary circulation is restored and right ventricular (RV) unloading is achieved (16). Anticoagulant and fibrinolytic therapies are crucial in managing thrombosis. Catheter-directed thrombolysis (CDT) has gained attention as an emerging technology. Studies have shown that CDT significantly reduces the right ventricular/left ventricular diameter ratio and lowers the incidence of adverse events or major bleeding in patients with moderate to high-risk acute PE (17). Compared with systemic fibrinolysis, CDT offers the potential advantage of enhancing thrombolysis through a synergistic effect of higher local fibrinolytic drug concentrations and mechanical thrombus disruption while minimizing the risk of major bleeding, especially intracranial hemorrhage (18). Following thrombolytic therapy, anticoagulation, and CDT, the patient's condition improved without any risk of bleeding.

## Conclusion

Protein S deficiency caused by mutations in the PROS1 gene is the genetic basis for this patient's pulmonary embolism. Thrombolytic therapy rapidly restores blood flow by promoting clot dissolution, while anticoagulant therapy maintains patency by preventing the formation of new clots. As an emerging treatment, catheter-directed thrombolysis is particularly suitable for patients requiring thrombolysis who are at an increased risk of bleeding.

## Data Availability

The original contributions presented in the study are included in the article/Supplementary Material, further inquiries can be directed to the corresponding author.

## References

[1] Goldhaber SZ. Pulmonary embolism. Lancet. (2004) 363(9417):1295–305. 10.1016/s0140-6736(04)16004-2 15094276

[2] Di Nisio M van Es N Büller HR. Deep vein thrombosis and pulmonary embolism. Lancet. (2016) 388(10063):3060–73. 10.1016/s0140-6736(16)30514-1 27375038

[3] Khan F Tritschler T Kahn SR Rodger MA. Venous thromboembolism. Lancet. (2021) 398(10294):64–77. 10.1016/s0140-6736(20)32658-1 33984268

[4] Castoldi E Maurissen LF Tormene D Spiezia L Gavasso S Radu C Similar hypercoagulable state and thrombosis risk in type I and type III protein S-deficient individuals from families with mixed type I/III protein S deficiency. Haematologica. (2010) 95(9):1563–71. 10.3324/haematol.2010.021923 20421270 PMC2930959

[5] Zeng T Li YI. Predicting RNA splicing from DNA sequence using pangolin. Genome Biol. (2022) 23(1):103. 10.1186/s13059-022-02664-4 35449021 PMC9022248

[6] Stefanucci L Collins J Sims MC Barrio-Hernandez I Sun L Burren OS The effects of pathogenic and likely pathogenic variants for inherited hemostasis disorders in 140 214 UK biobank participants. Blood. (2023) 142(24):2055–68. 10.1182/blood.2023020118 37647632 PMC10733830

[7] Xu F Zhou X Jin Y Yang L Pan J Wang M Analysis of PROS1 mutations and clinical characteristics in three Chinese families with hereditary protein S deficiency. Ann Hematol. (2024) 103(2):653–62. 10.1007/s00277-023-05607-6 38175252

[8] Beauchamp NJ Daly ME Makris M Preston FE Peake IR. A novel mutation in intron K of the PROS1 gene causes aberrant RNA splicing and is a common cause of protein S deficiency in a UK thrombophilia cohort. Thromb Haemost. (1998) 79(6):1086–91. 10.1055/s-0037-1615020 9657428

[9] Nagaya S Togashi T Akiyama M Imai Y Matsumoto H Moriya H Protein S deficiency caused by cryptic splicing due to the novel intron variant c.346+5G>C in PROS1. Thromb Res. (2023) 229:26–30. 10.1016/j.thromres.2023.06.020 37390525

[10] Mrożek M Wypasek E Alhenc-Gelas M Potaczek DP Undas A. Novel splice site mutation in the PROS1 gene in a Polish patient with venous thromboembolism: c.602-2delA, splice acceptor site of exon 7. Medicina (Kaunas). (2020) 56(9):485. 10.3390/medicina56090485 32971918 PMC7558706

[11] Okada H Kunishima S Hamaguchi M Takagi A Yamamoto K Takamatsu J A novel splice site mutation in intron C of PROS1 leads to markedly reduced mutant mRNA level, absence of thrombin-sensitive region, and impaired secretion and cofactor activity of mutant protein S. Thromb Res. (2010) 125(5):e246–50. 10.1016/j.thromres.2009.11.029 20022358

[12] Ding P Zhou Y Zhang KC Li S Long KL Chen J Case report: pROS1 (p.Leu584Arg) pathogenic mutation causes portal and superior mesenteric venous thromboembolism. Front Cardiovasc Med. (2023) 10:1277676. 10.3389/fcvm.2023.1277676 38034377 PMC10682651

[13] Gierula M Ahnström J. Anticoagulant protein S-new insights on interactions and functions. J Thromb Haemost. (2020) 18(11):2801–11. 10.1111/jth.15025 32702208

[14] Alshehri FS Bashmeil AA Alamar IA Alouda SK. The natural anticoagulant protein S; hemostatic functions and deficiency. Platelets. (2024) 35(1):2337907. 10.1080/09537104.2024.2337907 38602463

[15] Middeldorp S Nieuwlaat R Baumann Kreuziger L Coppens M Houghton D James AH American Society of hematology 2023 guidelines for management of venous thromboembolism: thrombophilia testing. Blood Adv. (2023) 7(22):7101–38. 10.1182/bloodadvances.2023010177 37195076 PMC10709681

[16] Millington SJ Aissaoui N Bowcock E Brodie D Burns KEA Douflé G High and intermediate risk pulmonary embolism in the ICU. Intensive Care Med. (2024) 50(2):195–208. 10.1007/s00134-023-07275-6 38112771

[17] Bashir R Foster M Iskander A Darki A Jaber W Rali PM Pharmacomechanical catheter-directed thrombolysis with the Bashir endovascular catheter for acute pulmonary embolism: the RESCUE study. JACC Cardiovasc Interv. (2022) 15(23):2427–36. 10.1016/j.jcin.2022.09.011 36121244

[18] Sadeghipour P Jenab Y Moosavi J Hosseini K Mohebbi B Hosseinsabet A Catheter-directed thrombolysis vs anticoagulation in patients with acute intermediate-high-risk pulmonary embolism: the CANARY randomized clinical trial. JAMA Cardiol. (2022) 7(12):1189–97. 10.1001/jamacardio.2022.3591 36260302 PMC9582964

